# The Profile and Characteristics of Young People Accessing Recently Implemented Community Forensic Child and Adolescent Mental Health Services (F:CAMHS) in Northern Ireland

**DOI:** 10.1007/s40653-024-00633-6

**Published:** 2024-03-28

**Authors:** Colm Walsh, Philip Anderson, Frances Caldwell

**Affiliations:** 1 Queen’s University, Belfast, UK; 2 Consultant Forensic Psychiatrist, SEHSCT, Belfast, UK; 3 SEHSCT, Belfast, UK

**Keywords:** Adolescents, Adversity, Forensic CAMHS, Mental Health, Trauma, Northern Ireland, Violence

## Abstract

Children under the age of 18 who are known to forensic child and adolescent mental health services often present with complex psychosocial and behavioural needs that are elevated compared with those in the general youth population. The Forensic Child and Adolescent Mental Health Service for Northern Ireland (FCAMHSNI) was commissioned in 2014 to support these children. Despite almost a decade of implementation, the profile and characteristics in the service remain under-analysed, impeding service improvement and making international comparisons more difficult. The primary aim of the current study was to address the regional gaps in how the needs of those accessing FCAMHSNI are understood. A secondary aim was to capture comparable data. Data on 107 accepted referrals are included in the analyses. The majority of cases within this time period were male (81.1%, *n* = 86) and the majority of presenting behaviours were related to violence and aggression 62.3% (*n* = 48). However, some forms of violence, such as harmful sexual behaviour, was relatively low when compared with other jurisdictions. Specific demographic characteristics such as gender and religious background appeared to be significant risk factors for referral to the service. Almost all of the sample are known to have experienced at least one potentially traumatic event (95.2%) and in more than one-third of cases, service users presented with co-morbid issues (35.6%, *n* = 37). These observations are discussed. This study adds to the growing international literature around the needs of forensically involved youth and helps to inform future service development and provision.

## Introduction

Whilst the concept of *‘high-risk’* is contested, concerns for the welfare of youth who come into contact with the justice system stretch back centuries (Hagell et al., 2004). More recent research illustrate the unmet needs and acute vulnerabilities (Dodge et al., 1990; Hindley et al., 2017; Olaghere et al., 2021); the range of trauma-related (Lansford et al., 2007; Baglivio et al., 2020), and the neurodevelopmental disorders (Hughes, 2012; Smith et al., 2022) that often co-occur with health harming behaviours (Lane et al., 2021) to place some youth at greater risk of offending (Dalsklev et al., 2019; Hales, 2019; Lane et al., 2021). Sociological, criminological and forensic research has found that youth engaged in the justice system have often been exposed to a range of adversities that are likely, in context, to contribute towards offending behaviour (Hindley et al., 2017; Dalsklev et al., 2019; Walsh et al., 2019).

The needs within the forensic population are complex, co-occurring, and elevated compared with the general population (Hagell et al., 2004; Lane. et al., 2021). Young people with additional neurodevelopmental disorders, or *‘neurodiversity’*, have additional vulnerabilities that enhance complexity. These young people are over-represented consistently within forensic or custodial services (Day, 2021; Kirby, 2021;). Neuro-diverse conditions include, but are not exclusive to, intellectual/learning disability; communication disorders; attention-deficit/hyperactivity disorder; and autism spectrum disorders. The reasons for the preponderance of neurodiversity in the justice system are complex and multifaceted.

Violence is a particularly problematic and pervasive form of adversity (Walsh & Gray, 2021) that when experienced more severely and more frequently can lead to trauma related pathology (Lader et al., 2003) and offending behaviour (Fowler et al., 2009) and exposure may be more common in communities that experience serious violence and conflict (Fowler et al., 2009).

For decades, Northern Ireland (NI) was intimately associated with violence, and despite the transition towards peace since 1998, exposure to violence and conflict remains high. Indeed, it remains the single most commonly experienced form of adversity among young people (Bunting et al., 2020), and as studies note, lifetime prevalence of exposure to violence is significantly above the EU average (McAloney et al., 2009; Hillis et al., 2016), with young men at particular risk of exposure and potential harm (Walsh & Schubotz, 2020; Walsh & Cunningham, 2023).

The rate of trauma related disorders such as PTSD is also comparatively higher in the NI context (Dalsklev et al., 2019). Factors such as living in communities characterised by high rates of violence (Fowler et al., 2009), a lack of adequate social support (Walsh, 2023), and a lack of adequate mental health screening and treatment (Duffy et al., 2021) are predictive of trauma related pathology (Trickey et al., 2012) and can untreated increase the risk of offending (Hindley et al., 2017).

Public health approaches are increasingly common, comprehensive, and evidence-based responses that seek to understand and address the root causes of serious harm, including violence within communities (Mercy et al., 1993; WHO, 2002). This type of approach recognizes that the acute needs presented by some youth are often complex and multi-dimensional, and are influenced by a wide range of psychiatric, social, cultural, and environmental factors (Pepin et al., 2018). Public health responses broadly imply joined up services; community and academic engagement (Massetti & Vivolo, 2010; Snider et al., 2010); a specific focus on data (Meyer et al., 2008); identification of key risks (Hawkins, Catalano & Arthur, 2002); the implementation of evidence informed responses and evaluation (Pound & Campbell, 2015).

Public health approaches require coordinated responses that are stratified into three levels of intervention; primary, secondary and tertiary (Hales, 2019). As with all interventions relating to reducing offending (Middleton & Shepherd, 2018), adolescent forensic mental health research and services can be described across the three public health model levels (Hales, 2019). **Primary approaches** are preventative in nature and apply universal services and supports to the general population. **Secondary approaches** are more targeted in their implementation, isolating those with known risk factors for violence from those of the general population and attempt to engage them in specific actions with the aim of reducing risks and improving outcomes. **Tertiary approaches** target a population who have persistent and chronic needs and who require highly specialised services. Forensic child and adolescent mental health services [FCAMHS] are an example of a tertiary level support and referrals are often made to these services when the needs of youth have not been sufficiently met in other services (Smith et al., 2022). They are small teams that provide highly specialist support and advice to the network of professionals around youth with complex needs and who present a risk to themselves or others (Lane et al., 2021).

In general terms, FCAMHS provide three types of support: support, advice and consultation when other professionals have reasonable concerns regarding a young person; case coordination for young people engaged with multiple services; and direct clinical work for a small proportion of young people (Hindley et al., 2017).

The Forensic Child and Adolescent Mental Health Service for Northern Ireland (FCAMHSNI) was commissioned in 2014, following the development of similar FCAMHS for young persons in England, Scotland and Wales (Hindley et al., 2017). FCAMHSNI was primarily commissioned to support local children’s services with understanding and co-ordinating care for young person’s presenting with complex needs and significant risks of harm to others. It was also commissioned to have a regional gatekeeping role for referrals to secure CAMHS hospitals. Currently this is a service provision not available in Northern Ireland. FCAMHSNI is based in the South Eastern Health and Social Care Trust, but is a regional service providing support to all of the 5 health and social care trusts in Northern Ireland.

Despite being almost a decade since its inception, there has been no profile of the FCAMHSNI population thus far. Given the wider context, this lack of detailed insight into the complex needs of some of the most vulnerable youth in Northern Ireland limits wider responses, but it also limits the potential to compare the needs of this forensic population on an international basis.

The primary aim of the current study is to address the regional gaps in how the needs of those accessing FCAMHSNI are understood. Specifically, this study provides a regional picture of service activity and the characteristics of children accessing FCAMHSNI. A secondary aim is to present data that is comparable on a national and international level.

## Methods

Routine service data was collated from FCAMHSNI referrals between 5th April 2018 and 24th April 2023. The majority of data were collected from clinical entries, which included information gathered from referral forms at the point of entry to the service.

For the purposes of data collection, the team mirrored many of the variables collected by other FCAMHS teams in Great Britain (Lane et al., 2021). The data captured includes cases accepted as meeting FCAMHSNI referral criteria and opened as live cases, either Level 1 (liaison and advice), Level 2 (Consultation / direct assessment) or Level 3 (enhanced risk management).

Data analysed was taken from a standardised referral form as well as from the clinical consultations and assessment. Data includes details regarding the referral source, the reason/s for referral, current and previous engagement with clinical services including CAMHS, Youth Justice Agency, social services and others, known mental health difficulties and any diagnoses, and a range of demographic details (e.g., gender, age, educational status and social care status). Where data was captured but specific variables were missing, these were coded as ‘unknown’.

All children under the age of 18 accepted as meeting the FCAMHSNI referral criteria, whether for direct or indirect support were eligible for inclusion. Clinical data was screened and anonymised before being shared with the lead researcher.

Overall, data was received from 107 cases which represents that entire population engaged between 5th April 2018 and 24th April 2023. The most common sources of referral were from CAMHS teams (34%, *n* = 36) (see Table 1). This was followed by social services (29.2%, *n* = 31), secure care (17.9%, *n* = 19), and the Youth Justice Agency (17%, *n* = 18). A minority of referrals originated from other sources that were too low in frequency (< 5) to provide disaggregated details (1.9%, *n* = 2).

Approval for secondary data analysis was granted by the South Eastern Health and Social Care Trust’s Information Governance department for a service evaluation. Routine data was shared in line with standard data sharing agreements between the Trust and Queen’s University Belfast. Identifiers were removed prior to sharing. The anonymised dataset was encrypted and shared via a secure platform. Once decrypted, the dataset was stored on secure, web based cloud server managed by Queen’s University Belfast in line with international standards. From here, the data was coded and copied into a statistical programme for analyses (SPSS, V27).

A description of regional activity is presented. In order to address the primary aim of the study, a range of descriptive data is outlined. Whilst the primary aim of the study is to provide a profile of the population, significance tests are also reported to compare groups and enhance an understanding of the nuance within service delivery. Exploratory analyses include chi-square tests of independence, one-way Anova, and t-tests for independent groups to understand differences between groups disaggregated by variables such as gender, justice involvement and contact type.

## Results

### Sources of Referral

Across the entire sample, referrals to FCAMHSNI led to Level 1 indirect case input in 23 cases (24.2%). This involved liaison with professionals involved, provision of advice where relevant, followed by closure. In the majority of cases (*n* = 64, 67.4%) children were provided with Level 2 support which involved professional liaison, as well as a forensic consultation and/or specialised risk assessment where appropriate. In 8 cases (8.4%), children received Level 3 support which involved an initial consultation, assessment and enhanced risk management support. Excluding missing data (*n* = 20), a structured assessment was facilitated in 65.5% (*n* = 57) of cases. Cases which did not meet referral criteria were excluded from the data.

The age groups of the sample are presented in Table 1. On average, children referred to FCAMHSNI were 15.1 years old, however, this ranged between 8 and 17. A ${X}^{2}$ test of independence was carried out to explore potential associations between the ages of the children and the type of support provided. There was a statistically significant association between these variables, with younger children more likely to receive Level 3 support and older young people more likely to receive Level 1 (${X}^{2}$(8, *N* = 95) = 10.70, *p* = .02).

### Reasons for Referral

Overall, the majority of presenting behaviours were related to violence and aggression, which accounted for 62.3% (*n* = 48) of referrals (see Table 1). In more than one-in-ten cases (13%, *n* = 10), children were referred due to multiple offending behaviours. In 13% (*n* = 10) of cases, referrers were seeking a second opinion in a complex case. Fire setting accounted for 6.5% (*n* = 5) of cases. Harmful Sexual Behaviour (HSB) accounted for a minority of cases overall (3.9%, *n* = 3), and other behaviours that were not easily grouped and/or were in such low frequency, were captured under the heading of *‘other’* (1.9%, *n* = 2). To assess any statistically significant association between the reason for referral and FCAMHSNI input, a ${X}^{2}$ test of independence was carried out. There was no statistically significant association between these variables, with children no more or less likely to receive direct or indirect input regardless of the reasons for referral.

**Table 1:** Contact/referral source and reason for referral

		All cases	
		*N*	*%*
Age	8 or younger	1	0.9
	9–11	5	4.7
	12–15	41	38.3
	16 or older	59	55.1
	Unknown or missing	1	0.9
Referral source	CAMHS	36	34
	Social Care	31	29.2
	Youth Justice	18	17
	Secure	19	17.9
	Other	2	1.9
Reason for referral	Violence and aggression	48	62.3
	Multiple offences	10	13
	Harmful Sexual Behaviour	3	3.9
	Fire setting	5	6.5
	Other	1	1.3
	Second opinion in a complex case	10	13

### Agencies Involved and FCAMHSNI Input Duration

At referral, children were known to other agencies. At times, it was evident from the routinely collected data that some were involved with several agencies. For example, 79.2% (*n* = 84) of children and young people were known to mainstream CAMHS at the point of referral and 81.1% (*n* = 86) were previously known to mainstream CAMHS. In Northern Ireland, health and social care services are organised across five health and social care Trusts (see Table 2). There was variation across these five health Trust regions, with children least likely to be involved with CAMHS prior to referral in the South Eastern Health and Social Care Trust (70%) compared with those living in the Belfast (85.7%), Southern (76.9%), Northern (94.4%) and Western (78.3%) Health Trust areas. However, this was not at the point of statistical significance.

**Table 2:** Characteristics of children and young people referred to FCAMHSNI

		All cases	
		*N*	*%*
Gender	Male	86	81.1
	Female	20	18.9
Ethnicity	Black African	1	0.9
	Irish Traveller	6	5.7
	Mixed ethnicity	3	2.8
	Other Ethnic group	1	0.9
	White	95	89.6
Religion	Catholic	17	15.9
	Protestant	9	8.4
	Other	1	0.9
	Unknown/missing	80	74.8
Trust area	Belfast	21	20
	Northern	18	17.1
	Southern	13	12.4
	South Eastern	30	28.6
	Western	23	21.9
Social care involved	Total	99	93.4
	LAC	64	61
Justice involved	Current involvement	53	51
	Police involvement	62	58.5
	Youth Justice Agency	65	61.3
Justice involvement status	Bail	17	29.8
	Remand	7	12.3
	Sentenced	3	5.3
	Youth Conference	9	15.8
	Diversionary plan	4	7
	Other	3	5.3
Living arrangements	Secure care	16	15.2
	Custody	7	6.7
	Residential care	25	23.8
	Birth Family	30	28.6
	Open inpatient	8	7.6
	PICU	8	7.6
	Semi independent	3	2.9
	Low/Medium secure care	3	2.9
	Other family	4	3.8
	Foster care	1	1
Education status	Mainstream education	24	22.4
	NEET	28	26.9

Mean time from assessment to discharge was 138.64 days, ranging between 5 days and 534 days (SD = 86.66). There was a statistically significant difference in the mean duration (assessment to discharge) between the different reasons for referral *(F* (4, 70) = 2.5, *p* = .038). For instance, those referred with issues related to violence/aggression and those whose reasons for referral fell into the *‘other’* category, were significantly more likely to have a longer clinical input from FCAMHSNI. There were no statistically significant differences in mean duration based on gender, previous or current mainstream CAMHS involvement, educational or social care status, and criminal justice involvement. There was, however, a significant difference with regard to mental health issues. In particular, those with known PTSD issues (m = 252, SD = 89), were significantly more likely to spend greater time with FCAMHSNI support than those without (m = 138.9, SD = 85.27, t (93) = 1.87, *p* = .04). This was not the case for other mental health presentations (i.e., anxiety, depression, emotional dysregulation, low mood, psychosis, attachment, eating disorders, and co-morbid presentations). No cases required longer-term input of greater than one year.

### Characteristics of Children Accessing FCAMHSNI

Data illustrates a statistically significant difference in the gender of the sample in that the majority of cases within this time period were male (81.1%, *n* = 86), compared with less than one-in-five who were female (18.9%, *n* = 20) (${\text{X}}^{2}$(1, *N* = 106) = 41.1, *p* = < 0.001). Ethnically, the population were highly homogenous. Indeed, 89.6% (*n* = 95) self-identified as ‘white’, compared with only 5.7% (*n* = 6) who identified as *‘Irish Traveller’* and a combined 4.6% (*n* = 5) who identified as either Black African, Mixed Ethnicity or Other Ethnic Group. Despite the proportional underrepresentation of ethnic groups, these appear to be higher than that would be expected as a proportion of those in the NI population. Irish Travellers account close to 6% of this sample, they account for only 0.1% of the NI population (NISRA, 2022). Of those who provided data on religious background, 15.9% of the sample were from a catholic background compared with 8.9% who identified as protestant. Social Care status, educational status and Youth Justice status are outlined in Table 2. 93.4% (*n* = 99) of all of those referred were social care involved and 61% were Looked After Children (LAC). 51% (*n* = 53) of the sample were currently involved with the youth justice system. Specifically, 58.5% (*n* = 62) were involved with the police at the point of referral and 61.3% (*n* = 65) were involved with the Youth Justice Agency. Aligned with wider literature, 26.9% (*n* = 28) were not in education, training or employment at the point of referral.

A series of ${\text{X}}^{2}$ tests of independence using continuity correction were performed to explore associations between LAC status and other variables characteristic of the population. There was a statistically significant association between CSE ${\text{X}}^{2}$(1, *N* = 105) = 5.54, *p* = .019), multiple mental health concerns ${\text{X}}^{2}$(1, *N* = 104) = 4.55, *p* = .033) and LAC status. There were no other significant associations.

A series of ${\text{X}}^{2}$ tests of independence were performed to explore associations between current justice involvement and other variables characteristic of the population. There was a statistically significant association between those who were NEET and justice involvement ${\text{X}}^{2}$(1, *N* = 103) = 4.22, *p* = .04). Whilst males were more likely than females to be justice involved at the point of referral (52.4% v 45%), this was not at the point of statistical significance. Despite testing for a series of other associations (CAMHS involvement, any known mental health issues, multiple mental health issues, known issues with CSE) none of these were significant.

### Children’s Mental Health and Wellbeing at the Point of Referral

Routinely collected service level data illustrate that children referred to FCAMHSNI present with an array of mental health and wellbeing difficulties at the point of referral (see Table 3). Overall, it was established via the referral information that almost four-in-five children had a known mental health issue (78.8%, *n* = 82). These included anxiety, attachment difficulties, ADHD, autism spectrum disorder, conduct disorder, depression and low mood, eating disorders, emotional regulation difficulties, foetal alcohol spectrum disorder (FASD) and other neurodevelopmental disorders. In more than one-third of cases, children presented with co-morbid issues (35.6%, *n* = 37).

**Table 3:** Mental health or wellbeing presentation of children referred to community FCAMHSNI

		All cases	
		*N*	*%*
Presenting difficulties	Any known mental health issue	82	78.8
	Anxiety	49	47.1
	Attachment	16	15.4
	Depression/low mood	33	31.7
	Conduct disorder	1	0.9
	Eating disorder	2	1.9
	Attention Deficit Hyperactivity Disorder	43	47.8
	Autism Spectrum Disorder	18	20
	Foetal Alcohol Spectrum Disorder	4	4.4
	Intellectual disability	19	18.1
	Emotional dysregulation	2	1.9
	Neurodevelopmental disorder	60	56.1
	Post-Traumatic Stress Disorder	3	2.9
	Psychosis	15	14.4
	Multiple challenges	37	35.6
Trauma history	Total	100	95.2
CSE concerns*	Total	15	14
Structured assessment used	Total	57	65.5
CAMHS involvement	Current	84	79.2
	Previous	86	81.1

*CSE = Child Sexual Exploitation

Almost half of cases had ADHD, 18% had an intellectual disability and 20% had a diagnosis of autism spectrum disorder, reflecting trends previously highlighted that neurodevelopmental conditions have a high prevalence in forensic populations (Day, 2021).

Females were more likely to present with any, as well as with multiple mental health challenges compared with males (94.7%/52.6% v 75.3%/31.8%), however, neither of these were at the point of statistical significance. There was no statistically significant difference in the baseline mental health difficulties and the level of FCAMHSNI input. However, there was a statistically significant difference between those who initially presented with comorbid challenges and those who did not, and the level of input (${\text{X}}^{2}$(2, *N* = 94) = 9.98, *p* = .007). In other words, children with comorbid challenges were more likely to have direct input than those who did not. There was no statistically significant difference the mean age of young people and either single or multiple mental health challenges, possibly because the sample clustered around the ages of 15–17, thus reducing the potential to detect such differences. Neither was there any association between previous CAMHS contact and comorbidity, suggesting that for reasons beyond the scope of this paper, those from within this population with more acute mental health needs had not been supported with emerging difficulties at an earlier stage. The association between known CSE issues and mental health comorbidity was statistically significant ${\text{X}}^{2}$(1, *N* = 104) = 5.89, *p* = .015) indicating greater mental health difficulties for victims of sexual exploitation. This association was also found with regard to victims of CSE and specific mental health difficulties including, attachment ${(\text{X}}^{2}$(1, *N* = 104) = 6.1, *p* = .014) and eating disorder ${(\text{X}}^{2}$(1, *N* = 104) = 6.06, *p* = .014), but not for conduct disorder, emotional regulation, PTSD, depression, anxiety or psychosis. Children known to have CSE issues were more likely to be LAC (${\text{X}}^{2}$(1, *N* = 105) = 4.86, *p* = .05), but no more or less likely to be social care involved, justice involved or NEET than others in the population.

Almost all of the sample are known to have experienced at least one potentially traumatic event (95.2%). This compares with 37% who are estimated to be exposed to at least one adverse event in the general youth population (Bunting et al., 2020). The specific type of trauma (e.g., single incident accident, or developmental adversity such as abuse or neglect) was not captured and therefore further disaggregation is not possible. However, it does point to the link between trauma in young people and potential development of offending patterns, whilst also hinting at the utility of trauma screening and support at an earlier stage (Duffy et al., 2021). Because exposure to trauma appears to be so endemic across the sample, further associational analyses is not possible.

## Discussion

The outcomes of this study are the first of its kind to look at the characteristics of young people who are accepted into FCAMHS services in NI. While there are significant gaps in the data, the outcomes largely reflect those of previous studies and research more widely which has found a significant proportion of people who become involved in forensic services have a multiplicity of risks, needs, traumatic experiences and mental health challenges.

Many findings of this evaluation are in line with a recent review completed by FCAMHS services in NHS England (Lane et al., 2023), and the Wales Forensic Adolescent Consultation and Treatment Service (Kabelic et al., 2022). The majority of referrals to FCAMHSNI were for white males, reflecting trends found by Lane et al. (2023) and Kabelic et al. (2022). However, it is noteworthy that disproportionately high numbers of young people from the Irish Travelling community (5.7%) were referred to FCAMHSNI comparative to the relative Irish Traveller population, which was estimated at 0.1% in the 2021 Census (Equality Commission for NI, 2023). This is of particular interest as similar numbers were not identified in the other data sets.

A further finding which requires attention is that the available FCAMHSNI data suggests that there is over-representation of certain groups. For example, whilst Irish Travellers account for less than 6% of this sample, they account for only 0.1% of the NI population (NISRA, 2022). Thus, the proportion in this sample is actually more than 60 times higher than what could be expected if it reflected the actual population. Similarly, the religious characteristics of children appeared to disproportionally reflect some groups more than others. Despite missing data, there was an interesting observation that significantly more Catholics than Protestants accessed the service. In fact, the figure was 89.3% higher, and whilst it may be difficult to infer any statistically or clinically significant challenge on the basis of the numbers involved, they actually support observations of over-representation of Catholic youth in the youth justice system in Northern Ireland (McAlister et al., 2022). Interpretation of these findings should therefore be made with caution. However, it is notable that these findings mirror previous research which found that a majority of young people referred to the Youth Justice Agency in NI come from Catholic backgrounds (McAlister et al., 2022), and that in 2021/22, 51.9% of children in custody were Catholic; while 19.8% were Protestant (NISRA, 2022). Further statistics from adult populations in NI suggest similar trends, with the Police Service of Northern Ireland (PSNI) arresting and charging twice as many Catholics than Protestants between 2016 and 2021 (Winters, 2021). McAlister et al. (2022) suggest that it is material deprivation that contributes to increased rates of vulnerabilities (parental stress, educational disadvantage and fewer pro-social opportunities) that in turn creates criminogenic needs, and may therefore be one reason why we observe a similar clustering in F:CAMHS. Despite the well-established links between deprivation and crime, this alone cannot wholly explain the over-representation of Catholic youth in Northern Ireland-not least because Protestant youth also live in areas of elevated economic deprivation. While it is true that historically Catholic youth were more likely to live in areas of higher deprivation compared with protestant youth, the distance has narrowed, and while political identifies have remained stubbornly attached to religious lines, there has been a “…considerable weakening of ethno-religious identity as a meaningful dimension of inequality” (Flaherty & McAuley, 2023). Other factors may be at play, several of which are distinctly Northern Irish issues and a legacy of conflict. One hypothesis may be that it is in predominantly Catholic communities that trust in public services (e.g., police and health and social care) has increased, while the converse has happened in deprived and predominantly Protestant areas (Walsh & Schubotz, 2020). The implication is that despite being as likely to present with similar issues, those from Catholic backgrounds are more likely to be known to public services. Despite the observations and potential hypothesis, the over-representation of Catholics within forensic services is not well understood and such consistent findings in various areas of criminal justice and acute mental health require further research using designs that is adequately powered to identify the explanatory variables.

Lane et al’s (2023) large-scale study found that the majority of referrals (40%) came from CAMHS services, followed by social services. This in line with current findings from FCAMHSNI. However, Kabelic et al., (2022) found that 90% of FCAMHS referrals in Wales came from CAMHS services.

FCAMHSNI have a higher prevalence of Looked After Children being referred (61%) comparative to NHS England (30.1%), though overall, the majority of young people referred to both services were known to social care or had social services input, again highlighting the vulnerability of this population and increased exposure to developmental adversity including familial breakdown, abuse or neglect. Kabelic et al., (2022) noted a significant prevalence (29%) of referrals living in residential social services but looked after child status was not recorded.

This finding is particularly notable when evidence is considered which suggests that NI has the lowest proportionate rate of children being Looked After away from their families compared to England, Scotland or Wales (Bywaters et al., 2018). Given that NI has the highest documented poverty rates comparative to UK countries, it may be expected that it would have the highest numbers of young people in the care system, given that trends suggest the most deprived areas have up for four times more young people being Looked After than the least deprived areas (NI Assembly, 2016). Of more interest to this review, it may also be hypothesised that due to fewer young people being Looked After, fewer Looked After young people would be referred to forensic services comparative to NHS England. This does not appear to be the case given that there are double the (proportionate) number of youth being referred to FCAMHSNI who are LAC compared to NHS England. The reasons for this are unclear and require further consideration.

Furthermore, both FCAMHSNI and NHS England data found around a quarter of young people referred were not in education, employment or training, highlighting general instability, poor educational attainment and a potential lack of boundaries and support for these young people. This data was not available in the Welsh study.

FCAMHSNI’s referrals demonstrated a high level of trauma and adversity; 95% of referrals had a trauma history. There was also a significant degree of neurodiversity including one-in-five having a diagnosis of autism spectrum disorder (ASD); almost half having a diagnosis of attention deficit hyperactivity disorder (ADHD); and one-in-five having an intellectual disability. This largely reflects trends from forensic services in general (Day, 2021) and highlights the risk of young people with neurodevelopmental disorders becoming stigmatised, criminalised or ending up in the justice system which may not be conducive to their needs.

While FCAMHSNI did not gather data in relation to direct/indirect case input, anecdotal review would suggest that the majority of referrals to FCAMHSNI received indirect case input as the young person was not directly assessed or offered direct intervention by FCAMHSNI. In some cases, direct assessment may not be warranted due to a large number of services and professionals being involved and assessment or intervention being provided by alternative services such as CAMHS or Youth Justice Agency. The discrepancy between what is able to be provided by FCAMHS in England comparative to FCAMHSNI highlights there may be needs which are best met by specialist forensic services which cannot be met due to commissioning. This data was not available in the Welsh study.

Around 13% of FCAMHSNI cases were referred for the purpose of providing a second opinion to a complex case; this is comparative to 30% of NHS England referrals for the same reason. This data was not available in the Welsh study. This highlights a large difference in numbers of referrals for this reason that may suggest FCAMHSNI are not accepting referrals for a second opinion as frequently. This may be because other services are unaware it is an option to refer to FCAMHSNI for this reason. Alternatively, FCAMHSNI may be declining cases where risk criteria are not met yet where services could benefit from support and multiagency collaboration in managing young people presenting with complex needs and other high risk behaviours.

These data also illustrate the concerns that prompt referral to FCAMHS with more than three-fifths of all cases a result of violence and aggression related issues. This highlights the need for FCAMHS to sit alongside and nested within wider and more systemic public health structures in order to more fully prevent and respond to needs on a tiered basis ranging from universal provision right through to specialist and tertiary provision which the service currently provides.

Interestingly, this study highlighted significant deviation from previous studies with regard to harmful behaviours- specifically for harmful sexual behaviour (HSB) and fire-setting. FCAMHSNI received only 3% of its referrals for HSB and 6.5% for fire-setting; whereas Lane et al’s (2023) review found 30% of cases were referred for HSB and 7.8% for fire-setting. Kabelic et al. (2022) found that 51% of cases were referred for HSB and 25% for fire-setting. A possible explanation for the HSB discrepancy is the local commissioning arrangements; HSB services in NI are managed under social services rather than CAMHS management in each trust area, as is the case in NHS England. There may be scope to better improve working relationships and co-working of cases, particularly where HSB is a feature alongside other risk behaviours. NI and NHS England statistics were similar for fire-setting. However, the discrepancy between Wales and NI is not well understood and would require further evaluation.

The current study highlights some findings which differ from NHS England’s review. Of note, FCAMHSNI referrals have a significantly high proportion of young people with at least one traumatic event known (over 90%). This exceeds those found in FCAMHS England and in Wales. Furthermore, more LAC are referred to FCAMHS in NI.

In conclusion, Northern Ireland continues to recover from conflict and the legacy of trauma inducing experiences. Indeed, rates of trauma related disorders remain elevated compared with the rest of the UK (Bunting et al., 2012). FCAMHSNI was conceived and first implemented within this context and is unlikely to address the wide-ranging psychosocial implication alone. Evidence suggests that when nested within wider, collaborative, and evidence based public health frameworks, outcomes can be enhanced (Mercy et al., 1993; Snider et al., 2010). Whilst the utility of such approaches are not yet fully realised, this study adds to the evidence around complex needs of children and youth in Northern Ireland and how creates a basis on which to monitor progress and evaluate the impact of a range activities implemented at primary, secondary and tertiary levels (WHO, 2002; Hales, 2019).

### Limitations

It is a significant limitation of the evaluation that we don’t have a full and accurate measure of the trauma and adversity experienced by our young people in terms of types of adversities and accumulation of experiences. It would be of future interest to compile data on specific experiences of young people in NI which may differ from trends in terms of violence exposure or exploitation elsewhere; such as considering community threats such as paramilitarism, through which vulnerable young people may be easily exploited, harmed or exposed to organised crime or violence. The data also doesn’t provide a full insight into the nature of direct work completed in some cases which would assist in highlighting gaps.

The evaluation highlights a number of things which would be beneficial to further explore; the disproportionate number of young people from Catholic backgrounds remains poorly understood by is in line with statistics from Woodlands Juvenile Justice Centre which requires further explanation. It is unclear why more Catholic young people would be sent to custody or referred to forensic services. Comparative to FCAMHS in NHS England, FCAMHSNI do arguably less direct work with young people. In order to address this service gap, more resources in terms of practitioners would be required.

### Clinical Implications and Future Recommendations

NHS England data suggests their FCAMHSNI teams have direct input with around 1 in 4 of their referrals. Welsh FCAMHS data was not available but it was noted that direct contact was ‘rare’. FCAMHSNI have not had the relative capacity to provide NHS England equivalent input to our population due to its current commissioned staffing provision. This evaluation highlights the needs of our young people as being complex, multifaceted and requiring multiagency intervention. In terms of service provision, the data implies that there is a requirement for a robust staffing team within FCAMHSNI to provide direct assessment and intervention as appropriate for high risk young people. FCAMHSNI also received fewer cases for a second opinion on a complex case, perhaps because services were unaware that this was an option, or because, although complex, some cases may be rejected by FCAMHSNI for not meeting referral criteria. To address this gap, FCAMHSNI are developing a pilot regional clinic model in each trust area to begin in late 2023. This clinic model will provide a forum for practitioners in partner agencies to bring cases for discussion and support from FCAMHSNI, but which may not require FCAMHSNI to open them as referrals. It is envisioned this model will promote relationship building between relevant agencies, highlight FCAMHSNI as a regional service available to other services and ensure appropriate cases are being referred. In addition, there is an established pilot for a Youth Justice CAMHS practitioner operational in the South Eastern Trust. This was in response to identified needs of young people open to Youth Justice Agency who also had significant mental health needs and difficulties engaging with Step 3 CAMHS services. Both pilots may have offer an opportunity for the organisation of a more formalised regional child health network for young people with complex needs and significant risks of harm. It will be also important for these services to both be robustly linked to the new integrated mental health and enhanced therapeutic service in the regional secure care facilities, commissioned following the Review of Regional Facilities (2018).

From a data collection perspective, this study nods to the complexity of needs. However, the data is at best partial. Of note is the incomplete and underassessed area of psychological trauma. To inform service delivery and to provide greater insight into the complexity of needs, it is recommended that consideration be given to how the service could embed measure to capture exposure to potentially traumatic experiences, screen for stress-related responses to those experiences, and assess for clinically diagnosable disorders such as PTSD in a more routine way.

Longitudinal studies have consistently highlighted the importance of intervening early with young people at risk to prevent entrenched, multiple adverse outcomes, including poor mental health, suicide, substance misuse, unemployment, and offending. There is strong evidence that the most effective way to reduce both offending and other poor outcomes for young persons is to work with families whose children are at the highest risk, at the earliest point possible, particularly where children are showing early signs of behavioural problems (Khan, 2010).

Young persons presenting with problem behaviours have experienced significant childhood adversity and attachment difficulties. It is crucial the systemic context of their presentation is taken account of and addressed. Poor parenting and family dysfunction explains up to 30–40% of problematic behaviour in children, indicating a need to focus predominantly on strengthening parenting skills and on building the child’s resilience (Khan, 2010). There are interventions specifically aimed at reducing problem behaviours. These include parent training for parents of primary school children (Scott, 2005) and MST for older adolescents (Fonaghy, 2018). There is clear evidence of the potential long-term costs efficiencies of early intervention, with costs associated with a child with severe conduct disorder estimated at £70,000 per head. Emerging evidence of effective early intervention can therefore support significant economic savings (Hughes, 2012).
